# Acute Cardiomyopathy in a Prisoner on a Hunger Strike

**DOI:** 10.7759/cureus.51949

**Published:** 2024-01-09

**Authors:** Tenes J Paul, Glenn Stokken

**Affiliations:** 1 Cardiovascular Medicine, University of Massachusetts Chan Medical School, Worcester, USA

**Keywords:** stress-induced cardiomyopathy, takotsubo cardiomyopathy, sudden cardiac death, anorexia nervosa (an), acute myocardial injury, male prisoner, cardiac mri (cmr), heart failure with reduced ejection fraction, short-term starvation, hunger strike

## Abstract

Chronic starvation and its associated metabolic derangements are known to have dangerous cardiovascular implications in the long term, but less is known about the cardiovascular consequences of acute starvation, such as in the context of a hunger strike. This case describes a patient who presented with signs and symptoms of acute coronary syndrome which began two weeks into a hunger strike and was ultimately found to have stress cardiomyopathy with complete resolution on subsequent imaging.

## Introduction

Hunger striking is a common method of civil disobedience that has known health consequences affecting multiple organ systems. The cardiovascular effects of long-term starvation are well documented, including arrhythmias, heart failure, and, rarely, sudden cardiac death. Most commonly, cardiac disease in this population is subacute to chronic in onset, related to the gradual depletion of vitamin stores [1,2]. Prior case reports have described the development of stress cardiomyopathy and cardiogenic shock in patients with chronic starvation due to malnutrition in the setting of eating disorders, famine, gastrointestinal malignancies, and neuromotor disorders, primarily driven by refractory hypoglycemic coma [3]. Furthermore, the dangers of refeeding have been well documented, including the potential for cardiovascular collapse with fatal ventricular arrhythmias caused by shifting electrolytes including phosphorus, potassium, and magnesium [4]. Notably however, the vast majority of cases of starvation and malnutrition resulting in cardiovascular complications are in the context of chronic starvation [5] due to systemic, inflammatory diseases (such as malignancies) or non-inflammatory diseases (such as anorexia nervosa) [2,3,6-8]. Less is known about the cardiovascular complications of starvation that develop over a shorter time frame, such as in the case of a hunger strike.

In contrast to cases of chronic starvation due to malnutrition or eating disorders, acute starvation results in different effects on the cardiovascular and sympathetic nervous systems [9]. In acute starvation among hunger strikers, death often occurs before the severe degree of emaciation seen with chronic starvation [10]. The specific cardiovascular diseases associated with acute starvation from hunger strikes remain poorly defined. We present a case of acute cardiomyopathy that developed in the setting of a hunger strike.

## Case presentation

A 59-year-old male with a baseline body mass index of 22 kg/m^2^ as well as a history of spontaneous pneumothorax, mood disorder, prior hernia repair, and intermittent homelessness presented from his psychiatric facility with a chief complaint of non-radiating chest pressure, ongoing for two to three days. The chest pressure was not associated with exertion. He denied shortness of breath or orthopnea, but did endorse lightheadedness, particularly with position changes. He took no medications regularly and denied any family history of heart disease. Of note, the patient had begun a hunger strike 14 days prior. During his hunger strike, he had consumed only water, milk, and molasses.

**Figure 1:**
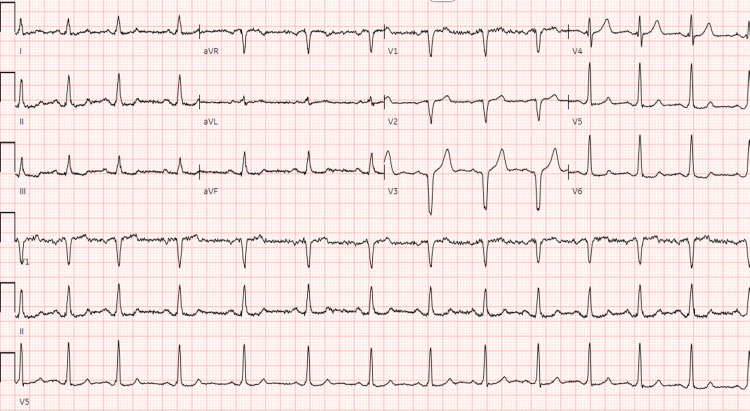
Initial electrocardiogram demonstrating sinus rhythm with Q waves in leads V1-V3

On initial examination, his heart rate was 74 beats per minute and regular, and his blood pressure was 113/80. Cardiac examination was unremarkable. Electrocardiogram (ECG) revealed sinus rhythm with Q waves in V1-V3 (Figure 1).

**Figure 2:**
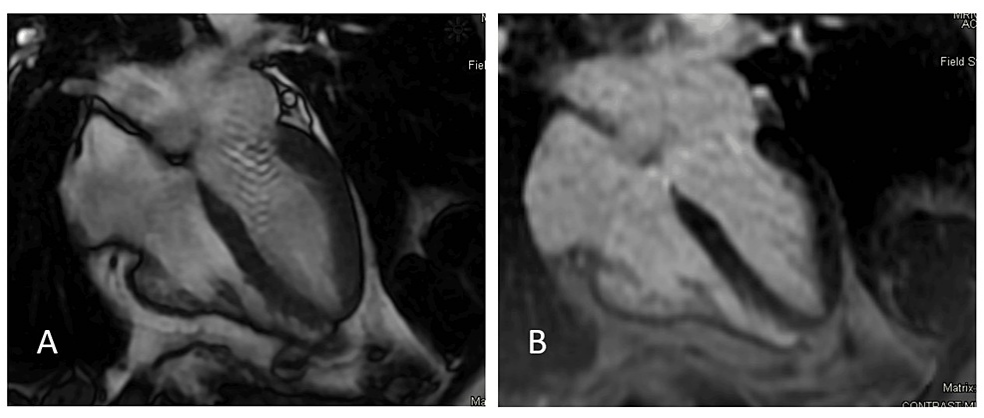
Cardiac MRI through the four-chamber view. Panel A shows a steady-state free precession frame in systole demonstrating normal left ventricular systolic function. Panel B demonstrates the corresponding phase-sensitive inversion recovery sequence showing a normal left ventricle with no evidence of late gadolinium enhancement

Laboratory data was significant for an elevated anion gap 16 (reference range: 5-15) with normal potassium, magnesium, sodium, glucose, and creatinine. Troponin I was elevated at 0.37 ng/mL (reference range: 0.01-0.04 ng/mL), decreasing to 0.35 ng/mL on subsequent check after three hours and 20 minutes. Low-density lipoprotein was elevated at 170 mg/dL. Prealbumin was 13 mg/dL (reference range: 18-40). Erythrocyte sedimentation rate (ESR), C-reactive protein (CRP), thyroid-stimulating hormone (TSH), folate, and B12 were normal (Table 1). He underwent a transthoracic echocardiogram which showed a normal-sized left ventricle with moderately reduced left ventricular ejection fraction of 40% by 3D volumetric analysis and hypokinesis of the mid-to-distal left anterior descending artery territory. His presentation was concerning for acute coronary syndrome. As such, he was treated with aspirin, atorvastatin, nitroglycerin, and intravenous heparin. His troponin trend remained flat, reducing the suspicion for a type I non-ST elevation myocardial infarction. He underwent exercise myocardial perfusion single-photon emission computed tomography (SPECT) imaging during which he completed 11 minutes of a standard Bruce protocol, remaining asymptomatic with no scan evidence of ischemia. For further characterization of the coronary anatomy, he underwent a coronary CT on hospital day 4 which showed a calcium score of 0. Out of concern for infiltrative or inflammatory processes, he underwent a cardiac MRI on hospital day 5 which showed that his left ventricular systolic function had improved, with an ejection fraction of 55% and no evidence of delayed gadolinium enhancement (Figure 2A, Figure 2B, Video 1). The diagnosis was presumed to be stress cardiomyopathy. The patient was discharged with recommendations for supportive care should he continue his hunger strike.

**Table 1:** Laboratory evaluation H: high; L: low; ESR: erythrocyte sedimentation rate; CRP: C-reactive protein; TSH: thyroid-stimulating hormone

Laboratory test	Value	Reference range
Sodium	135	135-145 mmol/L
Potassium	4.3	3.5-5.3 mmol/L
Creatinine	1.1	0.6-1.3 mg/dL
Magnesium	1.8	1.6-2.4 mg/dL
Glucose	71	70-99 mg/dL
Anion gap	16 H	5-15
Troponin I (initial)	0.37 H	0.01-0.04 ng/mL
Troponin I (subsequent)	0.35 H	0.01-0.04 ng/mL
Brain natriuretic peptide	6	<100 pg/mL
Low-density lipoprotein	170 H	<100 mg/dL
Triglycerides	85	<150 mg/dL
Prealbumin	13 L	18-40 mg/dL
ESR	5	<20 mm/hr
CRP	<1	<10 mg/L
TSH	0.612	0.280-3.890 µIU/mL
Folate	16.8	>5.9 ng/mL
Vitamin B12	376	182-803 pg/mL

**Video 1:** Cardiac MRI SSFP clip through the four-chamber view demonstrating normal left ventricular systolic function SSFP: steady-state free precession

## Discussion

The literature on cardiac disease and starvation is largely derived from studies of chronic malnutrition due to non-inflammatory diseases like anorexia nervosa. The cardiovascular impact of this group of diseases can be wide-ranging. The most common arrhythmia among these individuals is sinus bradycardia, often accompanied by orthostatic changes [6]. Regarding structural changes to the heart, prolonged cases of starvation can result in a significant reduction of left ventricular mass, up to 30-50%, often resulting in mitral valve prolapse. Protein deficiency and low thyroid hormone can result in mild pericardial effusions [2,6]. Chronic cardiomyopathy and heart failure can be seen, but the mechanism of these processes is due to the underlying etiology of chronic starvation. Deficiencies in vitamins like thiamine can result in wet beriberi [7]. Substances like ipecac, at times abused by patients with bulimia nervosa, can result in direct cardiotoxicity [8]. In cases of anorexia, premature death is most commonly from noncardiac causes.

In contrast to chronic malnutrition due to malignancy, anorexia nervosa, or vitamin deficiencies, which can often persist for years, the typical duration of a hunger strike can vary from two months to a year depending on baseline levels of protein and fat stores [1]. Among patients on hunger strikes, cardiovascular complications outside of arrhythmias are rare [1,2]. Prolongation of the QT interval can develop due to severe electrolyte abnormalities and, rarely, can cause torsades de pointes, ventricular fibrillation, and sudden cardiac death [2]. The development of cardiomyopathy has been reported among individuals with other forms of more chronic starvation, in certain cases resulting in cardiogenic shock and death [3,7,8]. However, the development of cardiomyopathy among hunger strikers or other cases of shorter-term starvation has not been reported in the literature. Our patient, only 14 days into his hunger strike, presented with clinical and laboratory evidence of myocardial injury as well as a new cardiomyopathy with reduced left ventricular systolic function. Ischemia was ruled out with coronary imaging and stress testing. Within days of resuming a diet, his cardiomyopathy resolved with a cardiac MRI three days after his initial echocardiogram showing no evidence of infiltrative process, myocarditis, scar, or infarction.

## Conclusions

The differential diagnosis for cardiomyopathy is broad and merits careful investigation to appropriately treat reversible causes. The thorough evaluation for the etiology of new cardiomyopathy should include imaging and laboratory assessment for the most common causes, with further investigation guided by the clinical history.

Although cardiomyopathy is a known complication of diseases that result in chronic malnutrition, this case demonstrates that the development of cardiomyopathy is also possible in the setting of short-term starvation caused by a hunger strike. Hunger strikers should be carefully evaluated both on initial presentation and throughout refeeding. Cases of myocardial injury and cardiomyopathy are rare among this population, but can result in serious, potentially deadly complications including heart failure, cardiogenic shock, arrhythmias, and death. Physicians should closely monitor strikers for evidence of these potentially dangerous complications. 
